# Effects of *Eucommia ulmoides* Oliver Extracts on Odontoblast Differentiation in Human Dental Pulp Stem Cells

**DOI:** 10.3390/cimb47100805

**Published:** 2025-10-01

**Authors:** Hye-Ock Jang, Ji-Min Ju, Soo-Kyung Bae, Da-Sol Kim, Hyung-Ryong Kim

**Affiliations:** 1 Department of Dental Pharmacology, School of Dentistry, Pusan National University, 49, Busandaehak-ro, Yangsan-si 50612, Gyeongsangnam-do, Republic of Korea; 2 Dental and Life Science Institute, School of Dentistry, Pusan National University, 49, Busandaehak-ro, Yangsan-si 50612, Gyeongsangnam-do, Republic of Korea; 3 Department of Pharmacology, College of Dentistry, Jeonbuk National University, Jeonju-si 54896, Jeonbuk-do, Republic of Korea

**Keywords:** dental tissue engineering, *Eucommia ulmoides* Oliver, human dental pulp stem cells, natural product, odontoblast differentiation, SMAD signaling pathway, tooth regeneration

## Abstract

*Eucommia ulmoides* Oliver (*E. ulmoides*), a traditional medicinal plant, has been widely used for its antioxidant and anti-inflammatory properties. However, its effects on dental tissue regeneration remain largely unexplored. In this study, we investigated the odontogenic potential of *E. ulmoides* extract in human dental pulp stem cells (hDPSCs). Cell viability was assessed using the cell counting kit-8 (CCK-8) assay, and antioxidant activity was evaluated via the DPPH radical scavenging method. Odontoblast differentiation was examined using Alizarin Red S (ARS) staining, real-time PCR, and Western blot analysis of key differentiation markers, including dentin matrix protein 1 (*DMP-1*) and dentin sialophosphoprotein (*DSPP*). Our results demonstrated that *E. ulmoides* extract enhanced mineralization and upregulated both gene and protein expression of odontoblast differentiation markers in a dose-dependent manner. Furthermore, signaling pathway analysis revealed that *E. ulmoides* extract activated the SMAD pathway while downregulating ERK and p38 MAPK phosphorylation during odontogenic differentiation. These findings suggest that *E. ulmoides* extract promotes odontoblast differentiation in hDPSCs and may serve as a promising natural agent for dental tissue regeneration. These findings further underscore its potential clinical relevance as a therapeutic candidate to enhance dental tissue repair and regeneration.

## 1. Introduction

Odontoblasts are post-mitotic cells located at the interface between dental pulp and dentin, where they are essential for the formation of primary and secondary dentin [1]. Loss or dysfunction of odontoblasts impairs dentin development, causes progressive dental tissue damage, and results in difficulties with mastication, thereby reducing patients’ quality of life [2]. Regeneration of odontoblasts is therefore a critical step in restoring normal oral function. Since the discovery in 2017 that glycogen synthase kinase-3β (GSK-3β) inhibitors can induce tooth regeneration [3], interest in developing novel strategies for dental tissue repair has increased.

*Eucommia ulmoides* Oliver (*E. ulmoides*), a deciduous tree of the family Eucommiaceae, is native to temperate and subtropical regions and is widely cultivated in Korea, China, and Japan [4]. Its bark has traditionally been used to strengthen muscles and lungs and to regulate blood pressure. Aqueous extracts have shown beneficial effects on hyperlipidemia and fatty liver disease and are often consumed as tea [5]. Phytochemical analyses have identified phenolics, flavonoids, lignans, iridoids, steroids, and triterpenes in its leaves and bark. Among these compounds, lignan glycosides such as glucopyranosides exhibit anti-obesity, anti-cardiovascular, and anti-hyperlipidemic properties [6,7]. Furthermore, *E. ulmoides* extracts have been reported to alleviate depression [8], improve reproductive and sexual function [9,10], prevent muscle atrophy related to central nervous system injury [11,12], and reduce the risk of osteoporosis [11]. Despite these systemic health benefits, studies on the potential of *E. ulmoides* for tooth regeneration remain limited.

Although *E. ulmoides* has been associated with systemic therapeutic effects, its role in odontoblast differentiation has not been fully clarified. The present study aimed to evaluate the antioxidative properties of *E. ulmoides* extract and its ability to promote odontoblast differentiation in human dental pulp stem cells (hDPSCs). Odontogenic differentiation was assessed using Alizarin Red S (ARS) staining, real-time PCR, and Western blotting of odontoblast-specific markers. In addition, signaling pathways associated with odontogenic induction were examined. The phytochemical profile of the extract was further analyzed by ultra-performance liquid chromatography coupled with tandem mass spectrometry (UPLC–MS/MS). Together, these findings demonstrate that *E. ulmoides* extract enhances odontoblast differentiation and may represent a promising natural candidate for dental tissue engineering.

## 2. Materials and Methods

### 2.1. Cell Culture

hDPSCs were purchased from Lonza (PT-5025; Basel, Switzerland). The cells were cultured in StemMACS^TM^ MSC Expansion Media Kit XF (Miltenyi Biotec., Inc., Somerville, MA, USA) under a humidified atmosphere containing 5% CO_2_ at 37 °C. The medium was changed every 3 days.

### 2.2. Reagents

DPPH (Santa Cruz Biotechnology, Santa Cruz, CA, USA), CCK-8 assay kit (Abbkine, Atlanta, GA, USA), DMSO, α-MEM, and FBS (Gibco, Thermo Fisher Scientific, Waltham, MA, USA) were employed in this study. Antibiotic–antimycotic solution and PBS were obtained from Biowest (Nuaillé, France), and RIPA lysis buffer was supplied by iNtRON Biotechnology (Seongnam-si, Republic of Korea). Primary antibodies against phospho-SMAD 1/5 and total SMAD 1/5 were purchased from Thermo Fisher Scientific. DMP-1, DSPP, osteopontin (OPN), and β-actin were purchased from Santa Cruz Biotechnology (Santa Cruz, CA, USA), and the peroxidase-conjugated secondary antibodies were purchased from Enzo Corp. (New York, NY, USA).

### 2.3. Cell Viability Assay

To evaluate the effect of *E. ulmoides* extract on hDPSCs, the cells were first seeded into a 96-well plate at 2 × 10^4^ cells/well, *E. ulmoides* extract was added at concentrations of 10, 25, or 50 μg/mL, and the cells were then cultured for 72 h. These concentrations were selected because treatment above 50 μg/mL markedly reduced cell viability to below 50%, making higher concentrations unsuitable for subsequent experiments. After culturing, 10 μL of CCK-8 in 100 μL of α-MEM without FBS was added, and the culture was incubated for 2 h at 37 °C in a 5% CO2 incubator. Absorbance was then estimated using an enzyme-linked immunosorbent assay reader (Tecan, Männedorf, Switzerland) at a wavelength of 450 nm.

### 2.4. Anti-Oxidation/Radical-Scavenging Activity

The DPPH radical scavenging assay was performed following the method of Barros et al. (2007) [13]. Absorbance was recorded at 517 nm, and the scavenging activity (%) was calculated using the following formula:

Scavenging activity (%) = [100 − {(AB − AS)/AB}] × 100,

where AB represents the absorbance of the DPPH control, and AS denotes the absorbance of the sample.

### 2.5. Odontogenic Differentiation

Odontogenic differentiation was induced by culturing the cells for 3 weeks in odontogenic medium (10% FBS, 0.1 mM dexamethasone, 10 mM β-glycerophosphate, and 50 mM ascorbic acid in α-MEM). Then, 2% ARS stain with a pH of 4.3 (Sigma-Aldrich, St. Louis, MO, USA) was used to estimate extracellular matrix calcification after 15 min. To quantify mineral deposition, 300 µL of 10% (*w*/*v*) cetylpyridinium chloride (CPC; Sigma-Aldrich), prepared in 10 mM sodium phosphate buffer (pH 7.0), was added to each stained well and incubated for 20 min at room temperature with gentle agitation to elute Alizarin Red S. The eluate was transferred to a 96-well plate, and absorbance was measured at 562 nm against a CPC buffer blank.

### 2.6. RNA Extraction and cDNA Synthesis

For total RNA extraction, human dental pulp stem cells were cultured in 6-well plates (1 × 10^6^ cells/well), and odontoblast differentiation began after the confluency reached full density. After 7 and 21 days, the cells were harvested, and total cellular RNA was isolated using FavorPrep™ Tri-RNA reagent (Favorgen, Ping-Tung, Taiwan) according to the manufacturer’s instructions. cDNA was synthesized from total RNA (2 μg) using Accupower RT PreMix (Bioneer, Daejeon, Republic of Korea). The cDNA synthesis process consisted of two steps. First, the RNA was incubated with oligo dT, and then the cDNA was synthesized under the following conditions: 42 °C for 60 min and RTase inactivation at 94 °C for 5 min. The reaction was stopped by heating at 70 °C for 15 min.

### 2.7. Real-Time Quantitative PCR (RT-qPCR)

Power SYBR Green PCR Master Mix reagent (Thermo Fisher Scientific) was used for the real-time qPCR. The cDNA was diluted (1:10) in Power SYBR Green PCR Master Mix. The RT-qPCR was performed in 20 μL reactions that contained 5–20 ng cDNA, 1 μL of each primer (10 pM), and 10 μL Power SYBR Green PCR Master Mix using an ABI 7500 Instrument (Applied Biosystems, Warrington, UK). The data were analyzed using ABI software, v 2.0.5. (Applied Biosystems, Warrington, UK) and the values were determined using the ∆∆Ct method. The RT-qPCR primer sequences used in this experiment are listed in Table S1.

### 2.8. Western Blot Analysis

Cells were lysed in RIPA buffer (Sigma-Aldrich, St. Louis, MO, USA) supplemented with a protease inhibitor cocktail (Roche, Indianapolis, IN, USA) and phosphatase inhibitors. Extracted proteins were resolved by SDS-PAGE and subsequently transferred onto PVDF membranes (Millipore, Bedford, MA, USA). Membranes were incubated with specific primary antibodies, followed by HRP-conjugated secondary antibodies. Protein bands were detected using an enhanced chemiluminescence kit (Pierce Biotechnology, Rockford, IL, USA) and visualized with an LAS-4000 imaging system (Fujifilm, Tokyo, Japan). The primary and secondary antibodies employed in Western blotting are summarized in Table S2.

### 2.9. Extraction of E. ulmoides

*E. ulmoides* was obtained from Gwangmyeongdang (Ulsan, Republic of Korea). To prepare the extract, 200 g of dried material was immersed in 2 L of 99.8% methanol and gently agitated once daily for three days. The crude extract was filtered through 185 mm filter paper and concentrated under reduced pressure in a water bath. The concentrate was then lyophilized using a freeze dryer (Labconco, Kansas City, MO, USA), yielding 11.9%. The resulting powder was stored at −20 °C and subsequently dissolved in DMSO for in vitro experiments.

### 2.10. LC-MS/MS Analysis

This method was established based on manufacturer-recommended protocols from SCIEX and Waters. Metabolomic profiling was performed using a ZenoTOF ™ 7600 mass spectrometer (SCIEX, Framingham, MA, USA) coupled with an ExionLC™ UHPLC system (AB SCIEX, Redwood City, CA, USA).

Chromatographic separation was carried out on a Waters ACQUITY UPLC HSS T3 column (2.1 mm × 100 mm, 1.8 μm) maintained at 40 °C, with an injection volume of 5 μL and a flow rate of 0.3 mL/min.

○ Positive ion mode: The mobile phase consisted of water with 0.1% formic acid (A) and acetonitrile containing 0.1% formic acid (B).
○ Negative ion mode: The mobile phase consisted of 5 mM ammonium formate in water (A) and acetonitrile with 5 mM ammonium formate (B).

The gradient program, applied in both modes, was as follows:

0–1 min, 5% B; 1–12 min, 5–95% B; 12–14 min, 95% B; 14–14.1 min, 95–5% B; and 14.1–17 min, 5% B for column re-equilibration.

Mass spectrometry was performed in information-dependent acquisition (IDA) mode with the following parameters: ion spray voltage, +4500 V (positive)/−4500 V (negative); curtain gas (CUR), 35 psi; ion source gas 1 (GS1), 55 psi; ion source gas 2 (GS2), 60 psi; temperature, 550 °C; and scan range, *m/z* 50–1250.

MS/MS spectra were acquired using a collision energy of 35 ± 15 eV. Zeno trap pulsing was enabled during acquisition to enhance MS/MS sensitivity. The total running time was 30 min per sample.

### 2.11. Statistical Analysis

Data in graphs are presented as mean ± SEM of *n* independent experiments, as indicated in the figure legends. Since the dataset did not conform to a normal distribution, comparisons between *E. ulmoides*-treated groups and controls were performed using the Wilcoxon matched-pairs signed-rank test. Statistical significance was defined as * *p* < 0.05, ** *p* < 0.02, and *** *p* < 0.01.

LC-MS/MS table values report *m/z*, retention time (RT, min), and peak area (a.u.) from representative runs; no dispersion statistics were applied to these identification data.

## 3. Results

### 3.1. Effects of E. ulmoides Extract on Cell Proliferation and Anti-Oxidant Activity

**Figure 1:**
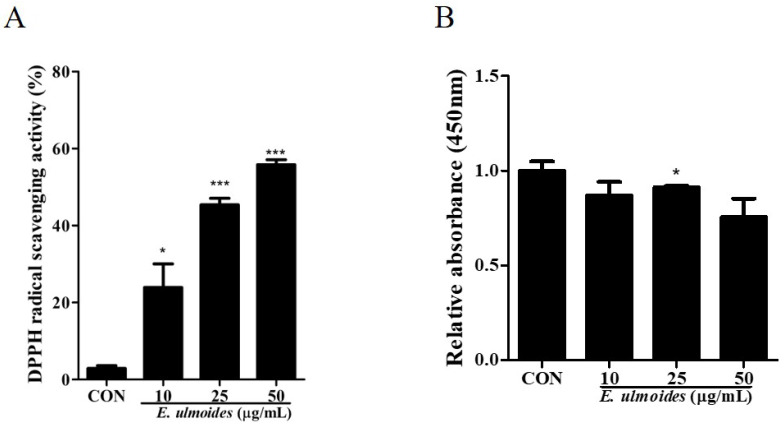
Antioxidant activity and the effects of *E. ulmoides* extract on cell proliferation. (**A**) A DPPH assay was performed, and the results indicated that the 10 µg/mL *E.ulmoides* extract-treated group showed a 20% increase in antioxidant activity. Antioxidant activity increased in a dose-dependent manner. (**B**) hDPSCs were incubated with 10, 25, or 50 µg/mL *E. ulmoides* extract for 72 h. Cell proliferation was measured using a CCK-8 assay. All data are presented as the mean ± SEM (*n* = 5). * *p* < 0.05, *** *p* < 0.01.

A DPPH assay was performed to examine the antioxidant activity of *E. ulmoides* (Figure 1A). At a concentration of 10 μg/mL, the extract increased radical scavenging activity by approximately 20-fold compared with the control. At 25 μg/mL, the activity was enhanced nearly 40-fold, demonstrating a stronger antioxidant effect. These findings clearly indicate that the increase in DPPH radical scavenging activity was dependent on the concentration of *E. ulmoides*. Overall, these results suggest that *E. ulmoides* extract exerts potent, dose-dependent antioxidant effects.

Cell proliferation was evaluated using the CCK-8 assay after 72 h of treatment with *E. ulmoides*. At first, the hDPSCs were treated with high concentrations of *E. ulmoides* (10, 25, and 50 μg/mL), and there were no toxic effects on the cells. The ideal *E. ulmoides* concentration was determined by testing several concentrations of *E. ulmoides* (10, 25, and 50 μg/mL). The cells were treated with *E. ulmoides* for 72 h, and the results showed that the various concentrations had no effect on cell proliferation (Figure 1B).

### 3.2. Effects of E. ulmoides Extract on Mineralization in hDPSCs

**Figure 2:**
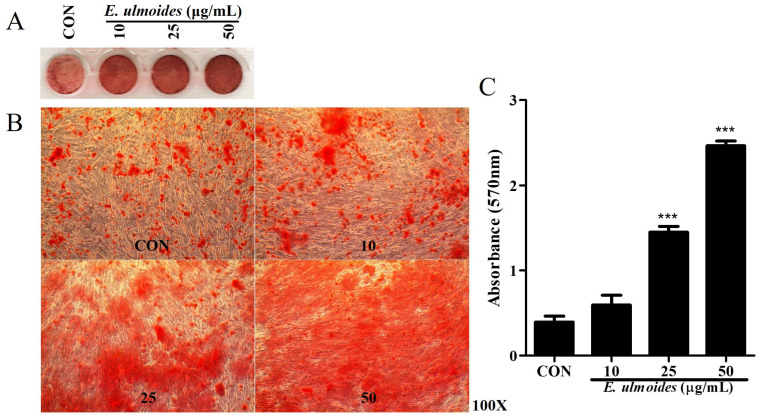
Effect of *E. ulmoides* extract on the odontoblastic differentiation of hDPSCs. (**A**) ARS staining of differentiated odontoblasts. (**B**) Representative microscope images showing differentiation. (**C**) Quantification of ARS staining. All data are presented as the mean ± SEM (*n* = 5). *** *p* < 0.01.

Figure 1 shows that the *E. ulmoides* extract was not toxic to hDPSCs. Therefore, we measured mineralization during *E. ulmoides* extract-treated odontoblast differentiation. Figure 2 shows that odontoblast differentiation was induced in hDPSCs. ARS staining was performed to evaluate mineralization. In Figure 2A, the ARS staining is shown by well plate scanning; each well was imaged using a microscope, and the quantification data are presented in Figure 2B. Quantitative analysis of staining intensity is displayed in Figure 2C.

At 10 μg/mL, *E. ulmoides* extract produced a slight increase in mineral deposition compared with the control. Treatment with 25 μg/mL resulted in a clear enhancement of mineralized nodule formation, while 50 μg/mL produced the most extensive mineralization, consistent with the highest absorbance values measured. These findings indicate that the extent of mineralization increased in proportion to the concentration of *E. ulmoides*.

In summary, ARS staining confirmed that *E. ulmoides* extract promoted odontoblast differentiation in hDPSCs in a dose-dependent manner.

### 3.3. Odontoblast Differentiation Markers Expression in E. ulmoides Extract-Treated hDPSCs

We evaluated the expression of odontoblast markers to determine whether *E. ulmoides* extract accelerates odontoblast differentiation. Alkaline phosphatase (*ALP*) and runt-related transcription factor-2 (*RUNX-2*) were used as the early markers. *DMP-1* and *DSPP* were also used in addition to these markers.

**Figure 3:**
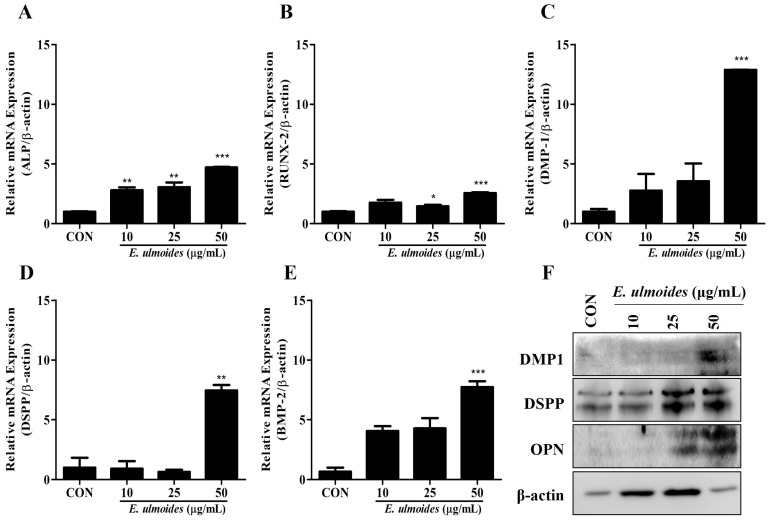
Effect of *E. ulmoides* extract on odontoblastic differentiation (OD) markers. (**A**–**E**) mRNA expression levels of various OD markers (*n* = 3). (**F**) Immunoblotting using antibodies against *OPN*, *DSPP*, and *DMP-1*. All data are presented as the mean ± SEM (*n* = 5). * *p* < 0.05, ** *p* < 0.02, *** *p* < 0.01.

As shown in Figure 3A–E, real-time PCR was performed to evaluate the pluripotency of *E. ulmoides* extract toward odontoblasts. At 7 days of induction, *ALP*, *RUNX-2*, and bone morphogenetic protein 2 (*BMP-2*) expression as early and middle odontoblast differentiation markers was measured, whereas *DMP-1* and *DSPP* expression was measured in the day 21 samples.

Western blotting was performed to evaluate OPN, DMP-1, and DSPP expression in day 21 samples, and the results showed that the *E. ulmoides* extract treatments upregulated the expression of these proteins (Figure 3F). These results indicate that *E. ulmoides* extract treatment upregulated odontoblast differentiation.

### 3.4. Signaling Pathway Analysis of E. ulmoides Extract-Treated Odontoblast Differentiation in hDPSCs

Mitogen-activated protein (MAP) kinases are an evolutionarily conserved family of serine/threonine kinases that constitute an interconnected signaling network. They play key roles in diverse cellular processes, including differentiation, apoptosis, and proliferation [2,14].

**Figure 4:**
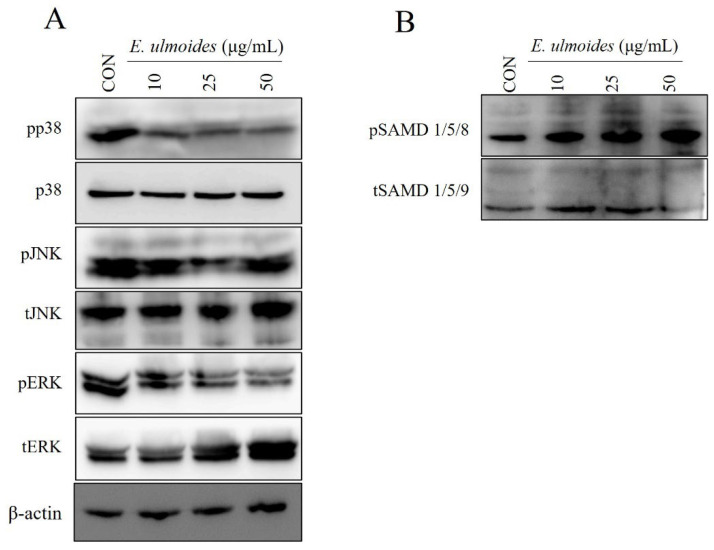
Effects of *E. ulmoides* extract on MAPK signaling pathways. hDPSCs were pre-treated with *E. ulmoides* extract (10, 25, or 50 μg/mL) for 30 min. Western blotting was used to detect phosphorylation levels of (**A**) phospho ERK, phospho JNK, and phospho p38, compared with their total protein levels. (**B**) Immunoblotting using antibodies against phospho SMAD 1/5/8 and total SMAD 1/5/9.

To investigate the role of MAPK signaling in *E. ulmoides*-mediated odontoblast differentiation, phosphorylation of MAPK proteins was assessed by Western blotting. Cells were pretreated as described above and subsequently exposed to *E. ulmoides* extract for 30 min during odontoblast induction. The results revealed that *E. ulmoides* extract reduced phosphorylation of ERK and p38 compared with the control (Figure 4A). Thus, treatment with *E. ulmoides* markedly suppressed p-ERK and p-p38 during odontoblast differentiation of hDPSCs. In contrast, phosphorylation of SMAD 1/5/8 was elevated following *E. ulmoides* treatment under odontogenic conditions (Figure 4B).

### 3.5. Quantitative and Qualitative LC-MS/MS Analysis of E. ulmoides

To confirm the presence of active ingredients in *E. ulmoides,* an LC-MS/MS analysis was performed using the following method. Table 1 shows the main components in the positive basic charge mode, and Table 2 shows the main components in the negative basic charge mode. Therefore, the qualitative analysis performed by LC-MS/MS is reliable.

## 4. Discussion

Dental health is closely related to nutrient intake, as it directly influences food consumption. Previous studies have shown a correlation between the number of natural teeth and human life expectancy [14]. This underscores the importance of maintaining oral health and forming good hygiene habits early in life. Still, once teeth are lost, therapeutic intervention becomes necessary. Dental implants and dentures are commonly used, but they cannot fully replace natural teeth and often present unresolved challenges, including biomechanical limitations and concerns about long-term stability [14].

The biological activity of *E. ulmoides* extract is likely related to its chemical composition. Compounds such as flavonoids, lignans, and iridoids are known to exert antioxidant effects, which can reduce oxidative stress in stem cells and create conditions favorable for differentiation. Oxidative stress has been shown to disrupt odontoblast differentiation by interfering with intracellular signaling, whereas antioxidants help restore redox balance and support lineage commitment. In this study, the extract displayed antioxidative activity alongside enhanced expression of odontoblast-associated genes and proteins, indicating that its phytochemical profile contributes to the observed cellular effects in hDPSCs [6,7,8].

To overcome the limitations of artificial replacements, we explored the potential of *E. ulmoides* extract to promote odontogenic differentiation in hDPSCs. *E. ulmoides* was chosen not only for its long history in herbal medicine but also because of reports suggesting regenerative effects on different tissues. In our study, methanol-based extraction was used to obtain a purified powder, which was applied to hDPSCs at 10, 25, and 50 μg/mL. Odontoblast differentiation was then evaluated by real-time PCR and western blotting of marker genes and proteins.

The results indicate that *E. ulmoides* extract enhances odontoblast differentiation and may be useful for dental tissue engineering. Comparable effects have been reported with other natural compounds. For example, resveratrol enhanced odontogenic differentiation of hDPSCs through SIRT1 and β-catenin activation [15]. Curcumin has been shown to promote mineralization via stimulation of the Wnt pathway [16], while green tea polyphenols such as EGCG increased odontoblast-like differentiation and mineral deposition [17]. These parallels suggest that *E. ulmoides* belongs to a broader class of plant-derived compounds with potential roles in dentin regeneration.

Mechanistically, our findings show that *E. ulmoides* extract modulates two major intracellular pathways associated with odontoblast differentiation. It suppressed ERK and p38 MAPK phosphorylation—commonly linked to cellular stress responses—while activating SMAD 1/5/8 signaling, a central pathway in odontogenic gene expression. Previous work has highlighted the importance of BMP2–SMAD signaling in odontoblast differentiation [18], and MAPK/ERK activity has also been recognized as a regulator of proliferation and differentiation [19]. This dual action indicates that *E. ulmoides* not only reduces inhibitory MAPK signals but also reinforces SMAD-mediated transcriptional activity, thereby promoting odontoblast lineage commitment.

LC-MS/MS analysis confirmed the presence of multiple bioactive molecules in the extract (Table 1 and Table 2). It is possible that these compounds act synergistically to produce the observed biological effects. Recent reviews emphasize that natural molecules, including flavonoids and lignans, can support dental pulp stem cell activity [20], and the phytochemical complexity of *E. ulmoides* itself has been well-documented [5]. Identifying the most active constituents, as well as establishing standardized extract preparations, will be important steps for translation into therapeutic use.

In summary, *E. ulmoides* extract promoted odontoblast differentiation in hDPSCs in a concentration-dependent manner and acted through modulation of MAPK and SMAD signaling pathways. Beyond its antioxidative and anti-inflammatory effects, the extract showed promise as a natural candidate for regenerative dentistry. These findings suggest that *E. ulmoides* extract could complement existing approaches not only in pulp and dentin regeneration but also in periodontal repair and preservation of pulp vitality. Strategies for physiological dentin regeneration are increasingly shifting toward biologically inspired approaches [21], and recent reviews highlight the feasibility of dental pulp regeneration for clinical application [22]. While our study was limited to in vitro experiments, further in vivo and preclinical studies will be required before clinical application can be realized.

## 5. Conclusions

In this study, *Eucommia ulmoides* extract was found to promote odontoblast differentiation of human dental pulp stem cells in a concentration-dependent manner. The extract enhanced the expression of odontoblast-related genes and proteins and influenced intracellular signaling by reducing ERK/p38 MAPK activity while activating SMAD 1/5/8 pathways. LC–MS/MS analysis revealed the presence of various phytochemicals, including flavonoids, lignans, and iridoids, which may contribute to these effects. Taken together, the findings indicate that *E. ulmoides* extract has potential as a natural agent for dental tissue regeneration. Further studies, including in vivo and preclinical experiments, will be needed to confirm these observations and to clarify the specific compounds responsible for the activity.

## Figures and Tables

**Table 1:** Representative metabolites identified from *E. ulmoides* extract using LC-MS/MS in positive ionization mode.

No.	Compound	*m/z*	RT (min)	Area (a.u.)	Score	Formula
1	L-Sorbose	203.0539	0.92	1,505,000	100	C_8_H_10_O_6_
2	Glycerophosphocholine	258.1117	0.89	209,000	98.9	C_7_H_19_N_3_O_5_S
3	Maltotriose	522.2058	0.96	339,000	95.8	C_5_H_20_N_20_O_9_
4	1-Kestose	543.1353	0.96	204,000	97.1	C_29_H_24_N_6_O_S_
5	6.α.-Mannobiose	325.1148	0.96	112,000	85.6	C_6_H_16_N_10_O_4_S
6	Stachyose	689.2149	0.96	165,000	81.8	C_18_H_32_N_20_O_4_S_3_
7	Loganic acid	399.1281	2.75	138,000	100	C_16_H_10_N_14_
8	Coniferyl aldehyde	179.0706	4	88,400	72.9	C_10_H_10_O_3_
9	Coumarin	147.0443	4.12	94,200	56	C_9_H6O_2_
10	Aspyrone	185.0811	4.1	171,000	83.8	C_9_H_12_O_4_
11	Swertiamarin	397.1138	4.79	354,000	90.5	C_19_H_24_O_5_S_2_
12	Catalpol	385.16	4.15	109,000	78.5	C_29_H_20_O
13	Tetrahydrocurcumin	355.1375	5.18	95,000	92.5	C_14_H_22_N_6_OS_2_
14	Phenylacetaldehyde	121.0653	4.79	51,000	88.6	C_8_H_8_O
15	Hydrocinnamic acid	151.0756	4.93	126,000	92	C_9_H_10_O_2_
16	Methyl trans-cinnamate	163.0755	5.46	30,000	94.7	C_10_H_10_O_2_
17	Anisaldehyde	137.06	4.72	25,600	51.5	C_8_H_8_O_2_
18	Eleutheroside E	760.3074	6.59	176,000	98.7	C_15_H_30_N_30_O_5_S
19	Aspyrone (replicate)	185.0811	7.03	78,000	87.6	C_8_H_8_O_3_
20	Swertiamarin (replicate)	385.16	4.16	109,000	78.5	C_29_H_20_O

*m/z*: mass-to-charge ratio; RT: retention time (min); Area: peak area (a.u.).

**Table 2:** Representative metabolites identified from *E. ulmoides* Oliver extracts using LC-MS/MS in negative ionization mode.

No.	Compound	*m/z*	RT (min)	Area (a.u.)	Score	Formula
1	Syringic acid	197.0488	2.35	50,000	89.1	C_6_H_14_O_5_S
2	Vanillic acid	167.0376	1.63	44,380	99.3	C_5_H_12_O_4_S
3	Geniposidic acid	373.1205	0.98	18,660	97.1	C_15_H_18_N_8_O_2_S
4	D-(+)-Raffinose	503.1698	2.01	30,370	93.7	C_26_H_20_N_10_O_2_
5	Hexadecanedioic acid	285.2107	9.23	76,360	100.0	C_14_H_30_N_4_S
6	Asperuloside	413.1502	3.87	103,600	97.1	C_14_H_18_N_14_S
7	Caffeic acid	179.0378	2.87	24,640	96.9	C_6_H_12_O_4_S
8	Chlorogenic acid	353.0938	1.02	14,000	100	C_7_H_12_N_14_O_5_
9	Aucubin	345.1250	4.03	67,020	96.2	C_6_H_14_N_14_O_4_
10	Melibiose	341.1149	0.97	93,380	99.2	C_11_H_18_N_8_O_3_S
11	Trehalose	387.1212	0.97	23,000	99.3	C_4_H_18_N_14_OS_2_
12	Sucrose	683.2365	0.97	138,000	54.6	C_18_H_18_N_2_O_5_
13	D-Glucose	225.0653	0.9	88,450	95.1	C_4_H_12_N_4_O_2_S
14	3-Furancarboxylic acid	253.1481	4.72	92,880	81.0	C_4_H_18_N_10_O_3_
15	Neochlorogenic acid	353.1652	5.60	103,600	95.0	C_8_H_22_N_10_O_6_
16	Genipin	225.0803	6.07	90,160	98.6	C_8_H_18_O_5_S
17	3-Hydroxy-9,10-dimethoxypterocarpan	299.0964	7.22	31,460	93.7	C_7_H_22_N_6_O_4_S_2_
18	(+)-Pinoresinol	357.1391	9.73	12,500	98.5	C_8_H_26_N_10_O_6_
19	Pinoresinol-glucoside	357.1395	7.60	71,910	99.5	C_3_H_20_N_16_O_4_S
20	3,5-Dicaffeoyl quinic acid	515.1226	26.19	264,400	100.0	C_7_H_12_O_6_

*m/z*: mass-to-charge ratio; RT: retention time (min); Area: peak area (a.u.).

## Data Availability

hDPSCs were purchased from Lonza (PT-5025; Basel, Switzerland). The original contributions presented in this study are included in the article/Supplementary Material. Further inquiries can be directed to the corresponding author(s).

## Supplementary Materials

The supporting information can be downloaded at: https://www.mdpi.com/article/10.3390/cimb47100805/s1.

## Abbreviations

*E. ulmoides*	*Eucommia ulmoides* Oliver
CCK-8	Cell counting kit-8
ALP	Alkaline phosphatase
RUNX-2	Runt-related transcription factor 2
OPN	Osteopontin
DMP-1	Dentin matrix acidic phosphoprotein 1
DSPP	Dentin sialophosphoprotein
ERK	Extracellular signal-regulated kinase
JNK	c-Jun NH2 terminal kinase
MAP kinase	Mitogen-activated protein kinase
SMAD	Mothers against decapentaplegic homolog
