# Supplementary material for: Effects of Eucommia ulmoides Oliver Extracts on Odontoblast Differentiation in Human Dental Pulp Stem Cells
Source: Curr Issues Mol Biol. 2025 Oct 1;47(10):805. doi: 10.3390/cimb47100805 (PMC12562457; doi:10.3390/cimb47100805)
Supplement: Supplementary file 1 [file cimb-47-00805-s001.zip › cimb-3853317-supplementary.pdf]

Supplementary Materials

Table S1. RT-qPCR Primer Sequences.

Gene	Forward Primer (5'→ 3')	Reverse Primer (5'→ 3')
DSPP	GAA GAT GCT GGC CTG GAT AA	TCT TCT TTC CCA TGG TCC TG
DMP1	ACC TTT GGA GAC GAA GAC AAT GGC	TGT CTT CAC TGG ACT GTG TGG TG
ALP	AGC TGA ACA GGA ACA ACG TGA	CTT CAT GGT GCC CGT GGT C
RUNX2	GGT TAA TCT CCG CAG GTC ACT	CAC TGT GCT GAA GAG GCT GTT
BMP-2	ACC CGC TGT CTT CTA GCG T	CTC AGG ACC TCG TCA GAG GG
β-actin	GGC ACC CAG CAC AAT GAA G	TGC GGT GGA CGA TGG AGG

Table S2. Western Blot Antibody Lists.

Protein	Catalog Number
DMP-1	SC-73633, Santa Cruz Biotechnology
DSPP	SC-73632, Santa Cruz Biotechnology
β-actin	SC-47778, Santa Cruz Biotechnology
Phospho-p38 MAPK (Thr180/Tyr182) Antibody	# 9211, Cell signaling
p38 MAPK Antibody	# 9212, Cell signaling
Phospho-SAPK/JNK (Thr183/Tyr185) (81E11)	#4668, Cell singnaling
SAPK/JNK Antibody	#9252, Cell singnaling
Phospho-p44/42 MAPK (Erk1/2)	# 4370, Cell singnaling
Total-ERK	p44/42 MAPK (Erk1/2), Cell singnaling
Phospho SMAD-1/5/8	AB3848-I, Merch Millipore
Total SMAD-1/5/9	PA5-80036, Thermofisher Scientific
goat anti-mouse IgG	sc-2004, Santa Cruz Biotechnology
goat anti-rabbit IgG	sc-2005, Santa Cruz Biotechnology
